# Importance of *IFT140* in Patients with Polycystic Kidney Disease Without a Family History

**DOI:** 10.1016/j.ekir.2024.06.021

**Published:** 2024-07-16

**Authors:** Takuya Fujimaru, Takayasu Mori, Akinari Sekine, Motoko Chiga, Shintaro Mandai, Hiroaki Kikuchi, Yutaro Mori, Yu Hara, Tamami Fujiki, Fumiaki Ando, Koichiro Susa, Soichiro Iimori, Shotaro Naito, Ryoichi Hanazawa, Akihiro Hirakawa, Toshio Mochizuki, Tatsuya Suwabe, Yoshifumi Ubara, Shinichi Uchida, Eisei Sohara

**Affiliations:** 1 Department of Nephrology, Graduate School of Medical and Dental Sciences, Tokyo Medical and Dental University, Tokyo, Japan; 2 Department of Nephrology, St Luke’s International Hospital, Tokyo, Japan; 3 Nephrology Center, Toranomon Hospital, Tokyo, Japan; 4 Okinaka Memorial Institute for Medical Research, Toranomon Hospital, Tokyo, Japan; 5 Clinical Laboratory, Tokyo Medical and Dental University Hospital, Tokyo Japan; 6 Department of Clinical Biostatistics, Graduate School of Medical and Dental Sciences, Tokyo Medical and Dental University, Tokyo, Japan; 7 PKD Nephrology Clinic, Tokyo, Japan

**Keywords:** ADPKD, *IFT140*, inherited kidney cystic disease, next-generation sequencing, polycystic kidney disease, total kidney volume

## Abstract

**Introduction:**

Recently, the monoallelic loss-of-function IFT140 variant was identified as a causative gene for autosomal dominant polycystic kidney disease (ADPKD). In patients with polycystic kidneys who have a positive family history, >90% have pathogenic variants in *PKD1* or *PKD2*, whereas only 1% have *IFT140*. However, approximately 40% of patients with polycystic kidneys without a family history do not have any pathogenic variants in *PKD1* and *PKD2*.

**Methods:**

We conducted a comprehensive genetic analysis of 157 adult patients with polycystic kidneys whose parents did not have evident polycystic kidneys. We sequenced up to 92 genes associated with inherited cystic kidney disease, including *IFT140*.

**Results:**

Of the 157 patients, 7 (4.5%) presented with monoallelic loss-of-function variants in the *IFT140* gene, 51 (32.5%) with pathogenic variants in the *PKD1* or *PKD2* gene, and 7 (4.5%) with pathogenic variants in other genes related to inherited kidney cystic disease. The proportion of monoallelic loss-of-function *IFT140* variants in this cohort was higher than that in previously reported cohorts with polycystic kidneys who had a positive family history. None of the patients with monoallelic loss-of-function *IFT140* variants had polycystic liver disease (PLD). Furthermore, patients with *IFT140* pathogenic variants had a significantly smaller kidney volume and a remarkably higher estimated glomerular filtration rate (eGFR) than those with *PKD1* pathogenic variants (*P* = 0.01 and 0.03, respectively).

**Conclusion:**

Because the phenotype of polycystic kidneys caused by the *IFT140* gene is mild, parental kidney disease may be overlooked. Therefore, patients without a positive family history are more likely to carry pathogenic variants in *IFT140*.

## Graphical abstract

**Figure:**
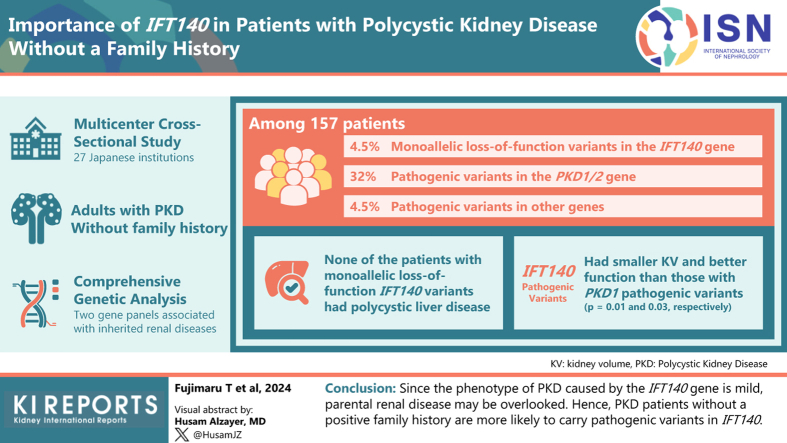

See Commentary on Page 2585

ADPKD is the most common inherited kidney disease, with a reported prevalence of 1:1000.^1^^,^^2^ ADPKD is characterized by the development and enlargement of kidney cysts with age, leading to end-stage kidney disease in most patients.^3^ ADPKD is genetically heterogeneous, with 2 major genes: *PKD1* (in approximately 78% of families) and *PKD2* (in approximately 15% of families).^4^ Recent whole-exome sequencing studies have identified pathogenic variants in genes such as *GANAB*, *DNAJB11*, and *ALG8* in a small proportion of patients with ADPKD.^5^ Furthermore, a monoallelic loss-of-function *IFT140* variant has been identified as the causative gene for ADPKD.^6^

*IFT140* encodes one of the subunits of the intraflagellar transport complex A, which is responsible for retrograde ciliary trafficking and ciliary entry of membrane proteins. Autosomal recessive diseases such as Mainzer-Saldino syndrome, a skeletal ciliopathy, are associated with *IFT140*.^7^

Despite a positive family history of polycystic kidneys typically being evident in ADPKD, in actual clinical scenarios, up to 25% of patients with ADPKD are identified without any positive family history of polycystic kidneys. This is because the affected parent either dies undiagnosed or is living with a mild, undetected form of the disease.^8^ Furthermore, reports indicate that approximately 40% of patients with polycystic kidneys who do not have a positive family history of polycystic kidneys do not present with *PKD1* or *PKD2* pathogenic variants.^9^, ^10^, ^11^ In patients with polycystic kidneys who have a positive family history of polycystic kidneys, ADPKD diagnosis is conducted using kidney imaging methods such as ultrasonography, computed tomography, or magnetic resonance imaging.^12^^,^^13^ Conversely, for patients without a family history of polycystic kidneys, there are no definitive imaging findings that conclusively diagnose ADPKD. Therefore, patients with polycystic kidneys without a family history of polycystic kidneys may carry gene variants associated with cystic kidney disease other than *PKD1* and *PKD2*, including *IFT140*. However, before the identification of *IFT140* as a causative gene of ADPKD, there have been only a few reports regarding the genetic characteristics of a cohort with polycystic kidneys without a family history of polycystic kidneys.^9^, ^10^, ^11^

In this study, we sequenced up to 92 genes associated with inherited cystic kidney disease, including *IFT140* in adult patients with polycystic kidneys whose parents do not have evident polycystic kidneys. We also studied the clinical characteristics of patients with pathogenic *IFT140* variants. This study reveals the specific genetic background of patients with polycystic kidneys without a family history of polycystic kidneys.

## Methods

### Patients

This is a multicenter cross-sectional study. From 2014 to 2023, adult patients with polycystic kidneys whose parents did not have evident polycystic kidneys were recruited from 27 Japanese institutions, including Tokyo Medical and Dental University. Polycystic kidneys were defined as having more than 5 cysts in each kidney, as detected by computed tomography or magnetic resonance imaging. In most cases, the presence of polycystic kidneys among the parents was determined via interviews only. Patients who had a parent or sibling with simple kidney cysts rather than polycystic kidneys or a child with polycystic kidneys were not excluded from the study. In addition, although patients with other inherited kidney diseases involving primary cilia may also exhibit the clinical symptoms of polycystic kidneys,^14^ in real-world clinical practice, they can be clinically distinguished from ADPKD based on complications in organs other than the kidneys and recessive inheritance. Therefore, we excluded patients with extrarenal complications such as retinitis pigmentosa indicative of nephronophthisis and liver fibrosis indicative of autosomal recessive polycystic kidney disease (ARPKD) and those aged under 20 years. However, patients with extrarenal complications of ADPKD, such as cerebral aneurysm and valvular heart disease, were included. This study, approved by the research ethics committee of Tokyo Medical and Dental University and other facilities, was conducted in accordance with the Declaration of Helsinki. All participants provided written informed consent.

Clinical data were gathered from medical records. eGFR was calculated using the Japanese glomerular filtration rate equation.^15^ Total kidney volume (TKV) was calculated from computed tomography or magnetic resonance imaging based on the volume of a modified ellipse for each kidney using the formula: volume = π/6 × length × width × depth.^16^

### Genetic Analysis

Comprehensive genetic testing was conducted using capture-based targeted next-generation sequencing. We analyzed 69 genes (panel version 1) or 92 genes (panel version 2) associated with inherited kidney cystic diseases such as ADPKD, ARPKD, nephronophthisis-related ciliopathy (including Joubert syndrome, Meckel syndrome, Senior-Løken syndrome, Bardet-Biedl syndrome, and skeletal ciliopathy), autosomal dominant tubulointerstitial kidney disease, autosomal dominant PLD, and other kidney cystic diseases (Table 1). The detailed methods are described in the Supplementary Methods and in previous reports.^9^^,^^17^^,^^18^ After filtering, all variants were evaluated by the American College of Medical Genetics and Genomics/Association for Molecular Pathology guideline.^19^ We defined "pathogenic" or "likely pathogenic" variants as pathogenic variants. Additionally, we extracted variants classified as variants of unknown significance (VUS) according to the American College of Medical Genetics and Genomics/Association for Molecular Pathology guidelines.^19^ To detect large genomic rearrangements, such as gross deletions or duplications, copy number variation analysis was conducted using Copy Number Analysis for Targeted Resequencing (http://contracnv.sourceforge.net/).^20^

**Table 1:** Disease categories and targeted genes included in the panels

Disease	Genes
ADPKD	*PKD1*, *PKD2*, *GANAB*^a^
ARPKD	*PKHD1*, *DZIP1L*^a^
Nephronophthisis	*NPHP1*, *INVS*, *NPHP3*, *NPHP4*, *IQCB1*, *CEP290*, *GLIS2*, *RPGRIP1L*, *NEK8*, *SDCCAG8*, *TMEM67*, *TTC21B*, *WDR19*, *ZNF423*, *CEP164*, *ANKS6*, *IFT172*, *CEP83*, *DCDC2*, *XPNPEP3*, *SLC41A1*, *MAPKBP1*^a^
JBS	*NPHP1*, *CEP290*, *RPGRIP1L*, *TMEM67*, *TTC21B*, *ZNF423*, *CEP164*, *IFT172*, *INPP5E*, *TMEM216*, *AHI1*, *ARL13B*, *CC2D2A*, *OFD1*, *KIF7*, *TCTN1*, *TMEM237*, *CEP41*, *TMEM138*, *C5orf42*, *TCTN3*, *TMEM231*, *CSPP1*, *PDE6D*, *MKS1*, *TCTN2*, *B9D1* *ARMC9*^a^, *CEP104*^a^, *CEP120*^a^, *KIAA0556*^a^, *KIAA0586*^a^, *PIBF1*^a^, *SUFU*^a^, *TMEM107*^a^
MKS	*NPHP3*, *CEP290*, *RPGRIP1L*, *TMEM67*, *TMEM216*, *CC2D2A*, *TMEM231*, *MKS1*, *TCTN2*, *B9D1*, *B9D2*, *KIF14*^a^, *TMEM107*^a^
SLS	*NPHP1*, *INVS*, *NPHP3*, *NPHP4*, *IQCB1*, *CEP290*, *GLIS2*, *SDCCAG8*, *WDR19*, *CEP164* *TRAF3IP1*^a^
BBS	*CEP290*, *SDCCAG8*, *TMEM67*, *TTC21B*, *WDR19*, *IFT172*, *MKS1*, *BBS1*, *BBS2*, *ARL6*, *BBS4*, *BBS5*, *MKKS*, *BBS7*, *TTC8*, *BBS9*, *BBS10*, *TRIM32*, *BBS12*, *WDPCP*, *BBIP1*, *IFT27*, *CCDC28B*, *C8orf37*^a^, *IFT74*^a^
Skeletal ciliopathy	*TTC21B*, *WDR19*, *IFT172*, *WDR35*, *IFT122*, *IFT140*, *IFT43*
ADTKD	*MUC1*, *UMOD*, *HNF1B*, *REN*^a^, *SEC61A1*^a^
ADPLD	*PRKCSH*^a^, *SEC63*^a^, *ALG8*^a^, *LRP5*^a^, *SEC61B*^a^, *GANAB*^a^
Others	*ASS1*, *NOTCH2*, *TSC2*^a^

ADPKD, autosomal dominant polycystic kidney disease; ADPLD, autosomal dominant polycystic liver disease; ADTKD, autosomal-dominant tubulointerstitial kidney disease; ARPKD, autosomal recessive polycystic kidney disease; BBS, Bardet-Biedl syndrome; JBS, Joubert syndrome; MKS, Meckel syndrome; SLS, Senior-Løken syndrome.

Gene panel version 1 included 69 genes, and version 2 included 92 genes.

a Genes included only in Gene Panel version 2.

### Statistical Analysis

We investigated genotype-phenotype correlations by comparing clinical characteristics among 3 groups: patients with *IFT140* pathogenic variants, *PKD1* or *PKD2* pathogenic variants, and those without any variants. The Kruskal-Wallis test was used to compare the medians of continuous variables in the 3 groups. For data yielding significant results from the Kruskal-Wallis test, a Bonferroni-adjusted Mann-Whitney U test was conducted to identify the significantly different groups. The Fisher exact test was used to compare the percentages of categorical variables. In addition, to evaluate the association between age and eGFR or TKV based on genetic characteristics (*IFT140* pathogenic variants, *PKD1* pathogenic variants, and *PKD2* pathogenic variants), analyses of covariance were performed with eGFR or TKV as the objective variable; and age, genetic characteristics, and the interaction term between age and genetic background as covariates. To prevent multicollinearity caused by the correlation between each variable and the interaction term, age was centered by subtracting the mean from each value. If the interaction term in the analyses of covariance model was not significant, the slopes of the regression lines between the groups were parallel. A significant difference was defined as a *P*-value < 0.05. All analyses were conducted using RStudio version 4.2.0. (RStudio Team [2020], RStudio: Integrated Development for R. RStudio, PBC, Boston, MA URL http://www.rstudio.com/).

## Results

### Eligible Patients

We investigated 157 adult patients with polycystic kidneys whose parents did not have any evident polycystic kidneys. No patients in this cohort were excluded because of retinitis pigmentosa or liver fibrosis. Among them, 53 patients were reported in our previous study.^9^ The clinical characteristics from the genetic analysis are shown in Table 2. The median age was 52 years. Of the 157 patients, 100 (63.7%) were male and 114 (81.4%) had hypertension. A liver cyst was found in 65 out of 93 patients (70.0%), and PLD, defined as having more than 20 cysts in the liver,^21^^,^^22^ was identified in 44 out of 93 patients (47.3%). The median eGFR was 43.8 ml/min per 1.73 m^2^ and the TKV was 1063 ml. Of 138 patients, 90 (65.2%) had TKV ≥ 750 ml.

**Table 2:** Clinical features at the time of genetic analysis in patients with adult patients with polycystic kidneys whose parents did not have evident polycystic kidneys

Features	All patients N = 157	*IFT140* pathogenic variants *n* = 7	*PKD1* or *PKD2* pathogenic variants *n* = 51	No variants *n* = 60	*P*- value
Age, yr	52.0 (44.0–61.0)	58.0 (49.0–63.0)	49.0 (42.5–54.5)	57.0 (47.8–67.8)	0.01
Male	100 (63.7)	6 (85.7)	33 (64.7)	39 (65.0)	0.60
Hypertension^a^	114 (81.4)	6 (85.7)	35 (81.4)	46 (83.6)	0.82
Liver cyst^b^	65 (70.0)	1 (14.3)	27 (100)	20 (57.1)	<0.001
PLD^b^ (>20 liver cysts)	44 (47.3)	0 (0)	21 (77.8)	12 (34.3)	<0.001
eGFR^c^, ml/min per 1.73 m^2^	43.8 (26.7–64.5)	58.0 (54.6–66.6)	48.7 (24.0–64.1)	42.9 (26.8–59.7)	0.23
TKV^d^	1063 (585–1839)	936 (612–2683)	1467 (975–2224)	832 (427–1645)	0.006
TKV^d^ ≥750 ml	90 (65.2)	4 (57.1)	41 (91.1)	29 (52.7)	<0.001

eGFR, estimated glomerular filtration rate; PLD, polycystic liver disease; TKV, total kidney volume.

Values are presented as median (interquartile range) or number (%). The comparison of 3 groups was analyzed using either Fisher’s exact tests or the Kruskal-Wallis test.

a Only 140 patients with available data were included: 7 patients with *IFT140* variants, 43 patients with *PKD1* or *PKD2* variants, 55 patients with no variants.

b Only 93 patients with available data were included: 7 patients with *IFT140* variants, 27 patients with *PKD1* or *PKD2* variants, 35 patients with no variants.

c Only 130 patients with available data were included: 7 patients with *IFT140* variants, 41 patients with *PKD1* or *PKD2* variants, 51 patients with no variants.

d Only 138 patients with available data were included: 7 patients with *IFT140* variants, 45 patients with *PKD1* or *PKD2* variants, 55 patients with no variants.

### Genetic Diagnosis

**Figure 1:**
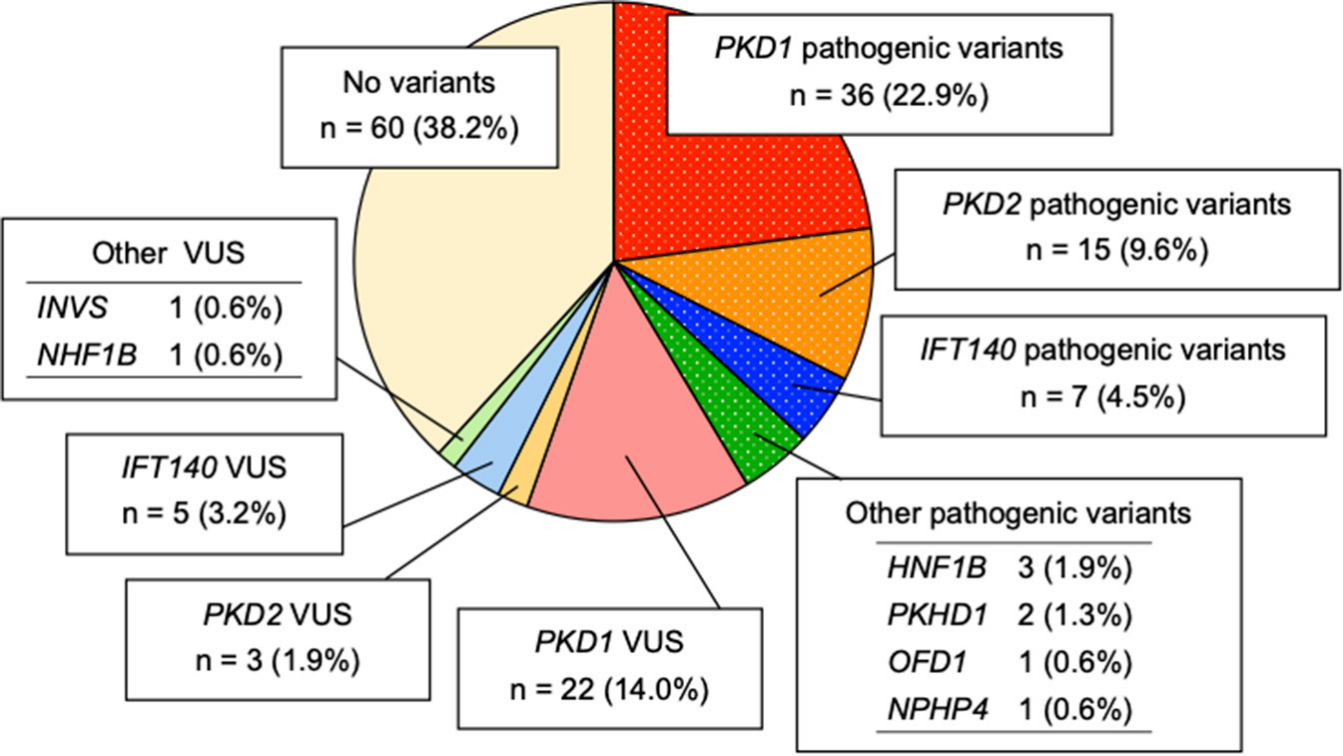
Variants in adult patients with polycystic kidneys whose parents do not have evident polycystic kidneys. Of the 157 patients, 36 (22.9%) had pathogenic variants in *PKD1*, 15 (9.6%) had pathogenic variants in *PKD2*, 7 (4.5%) had monoallelic loss-of-function variants in *IFT140*, and 7 (4.5%) had pathogenic variants in other genes. Three (1.9%) patients had *HNF1B* variants, 2 (1.3%) patients had *PKHD1* variants. One (0.6%) patient had *OFD1* variant, and 1 (0.6%) had *NPHP4* variants. VUS, variant of unknown significance.

In this study, 119 (78.8%) patients underwent genetic analysis using Gene Panel version 1, and 32 patients (21.2%) underwent genetic analysis using Gene Panel version 2. In Figure 1, we show the distribution of genetic diagnoses. Of 157 patients, 65 (41.4%) had pathogenic variants in genes associated with inherited kidney cystic disease. Monoallelic loss-of-function *IFT140* variants were detected in 7 patients (4.5%) (Table 3). *PKD1* and *PKD2* pathogenic variants were detected in 36 (22.9%) and 15 (9.6%) patients, respectively (Supplementary Table S1). Three patients (1.9%) had *HNF1B* pathogenic variants, 2 had *PKHD1* variants, and 1 each had variants in oral-facial-digital syndrome type 1 (*OFD1*) and *NPHP4* (Supplementary Table S2). In addition, 32 out of 157 patients (20.4%) had VUS in genes linked to inherited kidney cystic disease. *IFT140* VUS were detected in 5 patients (3.2%) (Table 4), *PKD1* and *PKD2* VUS in 22 (14.0%) and 3 (1.9%) patients, respectively (Supplementary Table S3), and VUS in other genes were found in 2 (1.3%) patients: 1 patient had it in *HNF1B* and 1 had it in *INVS* (Supplementary Table S4).

**Table 3:** Clinical features of patients with *IFT140* pathogenic variants

Patient ID.	Age^a^, yr	Sex	Hypertension	eGFR^a^, ml/min per 1.73 m^2^	TKV^a^, ml	Liver cyst^a^	Cerebral aneurysm^a^	Valvular disease^a^	Gene^b^	Variant	Zygosity	gnomAD^c^	ToMMo8.3K^d^	CADD^e^	ACMG classification	Reports
602^f^	78	M	+	52.7	5182	−	−	−	*IFT140*	c.1795dupA;p.Ile599fs	het.	none	none	33	P	none
723	58	M	+	58.0	935	−	−	−	*IFT140* *PKD1*	c.1795dupA;p.Ile599fs c.3449C>T:p.Pro1150Leu	het. het.	none 0.000008	none none	33 23.7	P VUS	none ^9^
730	42	M	+	84.7	653	−	+	−	*IFT140*	c.2500C>T;p.Arg834Ter	het.	0.000008	none	42	P	^6^^,^^23^^,^^24^
735	65	F	+	36.4	446	−	+	N/A	*IFT140* *PKD1*	c.439G>T;p.Glu147Ter c.1601C>T:p.Pro534Leu	het. het.	none 0.00002	none 0.003	38 23.1	P VUS	none none
942	53	M	+	68.8	3586	−	−	N/A	*IFT140*	c.1536_1539del;p.Lys512fs	het.	none	none	33	LP	none
1158^h^	61	M	+	56.5	1779	−	−	+^g^	*IFT140*	c.2068-2A>G	het.	0.000004541	none	33	P	^24^
1833	45	M	−	64.4	571	+ (No PLD)	+	N/A	*IFT140* *HNF1B*	c.2963del:p.Val988fs c.313G>A:p.Glu105Lys	het. het.	none none	none 0.0011	33 28.1	LP VUS	none 25, 26, 27

ACMG, American College of Medical Genetics and Genomics; CADD, Combined Annotation Dependent Depletion; eGFR, estimated glomerular filtration rate; F, female; het, heterozygous; LP, likely pathogenic; M, male; N/A, not available; P, pathogenic; PLD, polycystic liver disease; TKV, total kidney volume; het, heterozygous; VUS, variant of unknown significance.

a At the time of genetic analysis

b The following NCBI reference sequences were used: *IFT140*, NM_014714; *PKD1*, NM_001009944.

c Genome Aggregation Database, v2.1.1.^28^

d Allele frequency panel of 8,380 Japanese individuals from The Tohoku Medical Megabank Organization.^29^

e Combined Annotation-Dependent Depletion phred score.^30^

f The patient’s daughter had polycystic kidneys.

g Aortic stenosis.

h The patient’s mother had simple kidney cyst.

**Table 4:** Clinical features of patients with variants of unknown significance in *IFT140*

Patient ID	Age^a^, yr	Sex	Hypertension	eGFR^a^, ml/min per 1.73m^2^	TKV^a^, ml	Liver cyst^a^	Cerebral aneurysm^a^	Valvular disease^a^	Gene^b^	Variant	Zygosity	gnomAD^c^	ToMMo8.3K^d^	CADD^e^	ACMG classification	Reports
545	72	M	+	41.6	289	+ (PLD)	−	N/A	*IFT140*	c.359C>G:p.Ser120Cys	het.	none	0.001	24.4	VUS	none
619	56	M	+	KF	1415	−	N/A	N/A	*IFT140*	c.3602G>A:p.Arg1201his	het.	0.00006	0.005	23.5	VUS	none
761	55	F	+	40.5	1919	−	+	−	*IFT140*	c.1726C>T:p.Arg576Trp	het.	0.00003	0.0002	18.7	VUS	none
891	72	F	+	62.1	1453	+ (No PLD)	N/A	N/A	*IFT140*	c.1726C>T:p.Arg576Trp	het.	0.00003	0.0002	18.7	VUS	none
958	51	M	+	51.4	568	N/A	−	N/A	*IFT140*	c.1255G>A:p.Ala419Thr	het.	0.00001	0.0003	23	VUS	none

ACMG, American College of Medical Genetics and Genomics; CADD, Combined Annotation Dependent Depletion; eGFR, estimated glomerular filtration rate; F, female; het, heterozygous; KF, kidney failure; M, male; N/A, not available; PLD, polycystic liver disease; TKV, total kidney volume; VUS, variant of unknown significance.

a At the time of genetic analysis.

b The following NCBI reference sequences were used: *IFT140*, NM_014714.

c Genome Aggregation Database, v2.1.1.^28^

d Allele frequency panel of 8,380 Japanese individuals from The Tohoku Medical Megabank Organization.^29^

e Combined Annotation-Dependent Depletion Phred Score.^30^

Of 157 patients, 7 had parents with simple kidney cyst (1 with the *IFT140* pathogenic variant, 3 with the *PKD1* or *PKD2* pathogenic variant, 1 with the *OFD1* pathogenic variant, and 2 without pathogenic variants). In addition to these 7 patients, 1 with the *PKD1* pathogenic variant had a sibling with simple kidney cyst. Further, there were 2 patients whose children had polycystic kidneys. (1 with the *IFT140* pathogenic variant, 1 with the *PKD1* VUS). However, these families could not undergo genetic analysis.

### Clinical Findings of Patients with Pathogenic Variants and VUS in *IFT140*

As shown in Table 3, of the 7 patients with the *IFT140* pathogenic variants, 6 were men, and 6 had hypertension. Age at the time of genetic analysis ranged from 42 to 78 years, and the eGFR ranged from 36.4 to 84.7 ml/min per 1.73 m^2^. One patient had a history of gross hematuria (patient ID: 1158). In addition, 1 patient had liver cysts, and none had PLD. Three patients had cerebral aneurysms, and 1 had aortic stenosis. As shown in Table 4, 2 of the 7 patients with the *IFT140* VUS variant had liver cysts, and 1 of them presented with PLD. In addition, 1 patient had a cerebral aneurysm.

**Figure 2:**
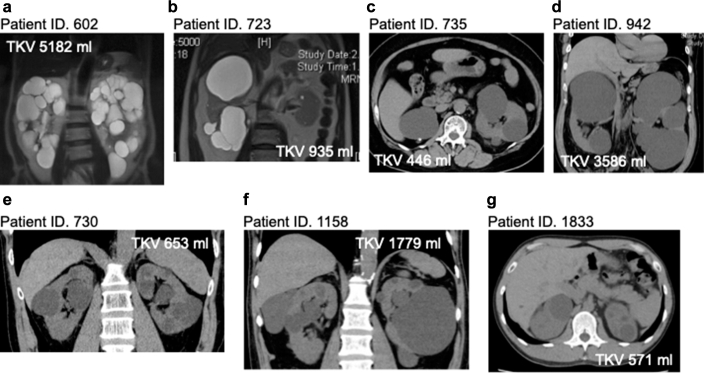
Computed tomography or magnetic resonance images of the kidneys in patients with pathogenic variants in *IFT140*. The TKV varied among these patients. Regarding kidney cysts, each cyst was larger than typical ADPKD, with some cases being asymmetrical. (a) Patient ID.602. (b) Patient ID. 723. (c) Patient ID.735. (d) Patient ID. 942. (e) Patient ID.730. (f) Patient ID. 1158. (g) Patient ID. 1833. ADPKD, autosomal dominant polycystic kidney disease; TKV, total kidney volume.

In Figure 2, we show computed tomography or magnetic resonance images of the kidneys in patients with pathogenic variants in *IFT140*. The TKV varied among these patients. Concerning kidney cysts, each one was larger than those typically found in ADPKD, with some cases being asymmetrical (Figure 2b). According to the Mayo Imaging Classification (MIC),^31^, numerous cases were in class 2A.

### Comparing Clinical Characteristics by Gene

We examined genotype-phenotype correlations by comparing the clinical characteristics of 3 groups, excluding patients with VUS: those with *IFT140* pathogenic variants, *PKD1* or *PKD2* pathogenic variants, and no variants (Table 2). Given the significant differences in age and TKV observed in the Kruskal-Wallis test (*P* = 0.01 and 0.006, respectively), we conducted a Bonferroni-adjusted Mann-Whitney U test. The Bonferroni-adjusted Mann-Whitney U test revealed that patients with *PKD1* or *PKD2* pathogenic variants were significantly younger (*P* = 0.008) and had significantly larger TKV (*P* = 0.005) than those without any variants. Furthermore, the distribution of liver cysts and PLD significantly differed among the 3 groups. Interestingly, only 1 patient with pathogenic *IFT140* variants had liver cysts, but none had PLD. Although there was no significant difference in eGFR between the 3 groups by the Kruskal-Wallis test, patients with pathogenic *IFT140* variants had the highest eGFR (Table 2).

**Figure 3:**
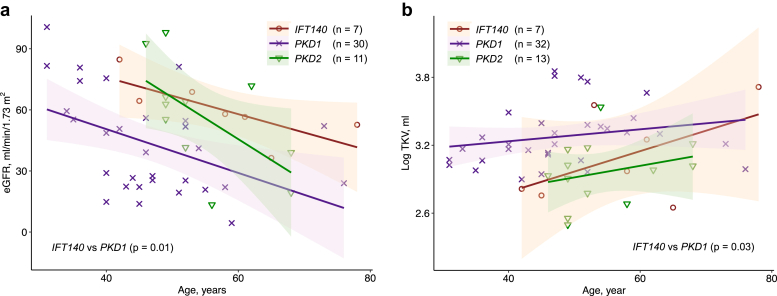
Distribution of age, eGFR, and TKV in patients with pathogenic variants in the *IFT140*, *PKD1*, or *PKD2* gene. Using the ANCOVA model, patients with pathogenic variants in the *IFT140* gene had a significantly higher eGFR and a smaller logarithmized TKV than those with pathogenic variants in the *PKD1* gene (*P* = 0.01 and 0.03, respectively). The shading indicates the 95% confidence intervals. (a) Scatter plot of age and eGFR. The regression equation obtained from a simple regression analysis was y = 112−0.903x for the *IFT140* group, y = 93.6−1.08x for the *PKD1* group, and y = 168−2.05x for the *PKD2* group. (b) Scatter plot of age and TKV. The regression equation obtained from a simple regression analysis was y = 2.07−0.018x for the *IFT140* group, y = 3.02−0.00527x for the *PKD1* group, and y = 2.4−0.0102x for the *PKD2* group. ANCOVA, analysis of covariance; eGFR, estimated glomerular filtration rate; TKV, total kidney volume.

In Figure 3, we show the scatterplots of age versus eGFR and age versus logarithmized TKV in patients with pathogenic variants in the *IFT140*, *PKD1*, or *PKD2* genes. Based on analyses of covariance, all interaction terms between centralized age and genetic background were not significant, and parallelism of the regression lines was assumed. In addition, patients with the pathogenic variant in the *IFT140* gene had a significantly higher eGFR and a smaller logarithmized TKV than those with the pathogenic variant in the *PKD1* gene (*P* = 0.01 and 0.03, respectively).

## Discussion

In this study, we sequenced up to 92 genes associated with inherited cystic kidney disease, including *IFT140,* in 157 adult patients with polycystic kidneys whose parents did not have evident polycystic kidneys. The analysis revealed that 7 of 157 patients (4.5%) had monoallelic loss-of-function *IFT140* variants. In cases with pathogenic variants in *IFT140*, kidney cysts were asymmetrical, with many atypical instances of 1 large cyst. In addition, all patients had higher eGFR and no PLD.

To the best of our knowledge, this study is the first to focus only on cases of polycystic kidneys without a positive family history of polycystic kidneys and to identify the subclinical rate of *IFT140*. We compared the proportion of monoallelic loss-of-function *IFT140* variants between the general ADPKD cohorts and cases without a positive family history of polycystic kidneys (Table 5). Senum *et al.*^6^ performed a genetic analysis on families diagnosed with ADPKD who were naive to genetic testing (*n* = 834) or who did not present with the *PKD1* and *PKD2* pathogenic variants (*n* = 381). Of the previously unscreened families, 16 of 834 patients (1.9%) had a monoallelic *IFT140* loss-of-function variant. On the other hand, of the 381 families that did not have pathogenic variants in the *PKD1* or *PKD2* genes in previous genetic analyses, 21 had pathogenic variants in the *IFT140* gene. Chang *et al.*^23^ conducted a genetic analysis of 235 patients clinically diagnosed with ADPKD. They found that 161 patients (68.5%) had possible pathogenic variants in the *PKD1* or *PKD2*, and 2 patients (0.9%) presented with truncation variants of *IFT140*. In contrast, our study only performed a comprehensive genetic analysis for adult patients with polycystic kidneys whose parents did not have evident polycystic kidneys. Interestingly, only 51 patients (32.5%) had pathogenic variants in *PKD1* or *PKD2*, whereas 7 patients (4.5%) had monoallelic loss-of-function *IFT140* variants (Figure 1). These results suggest that cases of polycystic kidneys without a positive family history of polycystic kidneys have fewer *PKD1* or *PKD2* pathogenic variants than those with a positive family history. Furthermore, *IFT140* VUS were identified in 5 patients (3.2%) in our study (Table 4). These variants exhibited very low allele frequencies and high Combined Annotation Dependent Depletion scores,^30^, which indicate the potential deleterious nature of the variants. Consequently, these variants meet the criteria for PM2 and PP3 as per the American College of Medical Genetics and Genomics guidelines.^19^ If the same amino acid variant is reported to be disease-causing, it satisfies PS1 and is categorized as a "likely pathogenic" variant. Therefore, the proportion of patients with pathogenic variants in *IFT140* is particularly high and cannot be ignored especially in the case of being limited to the cases without a positive family history of polycystic kidneys.

**Table 5:** Percentage of monoallelic loss-of-function *IFT140* variants in general polycystic kidneys cohorts and cases without a positive family history of polycystic kidneys

Study	Number of the patients with polycystic kidneys	Number of the patients with a monoallelic loss-of-function IFT140 variants	Number of the patients with a pathogenic variant in PKD1 or PKD2	Country
Senum. 2022.^6^	834 families^a^	16 families (1.9%)	N/A	Various countries
Chang. 2022.^23^	235 patients	2 patients (0.9%)	161 patients (68.5%)	USA
Present study	157 patients (included only cases without a positive family history of polycystic kidneys)	7 patients (4.5%)	51 patients (32.5%)	Japan

N/A, not available.

a Previously unscreened families only.

In our study, in the scatter plot of age and TKV, patients with the *IFT140* variants had a significantly smaller kidney volume than those with the *PKD1* variants (Figure 3b). Furthermore, in patients with *IFT140* pathogenic variants, each kidney cyst was larger than those typically seen in ADPKD, with some patients exhibiting asymmetrical kidney cysts (Figure 2). In addition, only 1 patient had liver cysts, but none had PLD (Table 2). In a previous study on ADPKD genetic analysis, patients with *IFT140* pathogenic variants frequently exhibited kidney enlargement. However, some patients exhibited atypical patterns such as asymmetric kidney cysts or microcysts.^6^ In the same study, out of 66 patients with *IFT140* pathogenic variants, only 9 (14%) had liver cysts and 2 (3%) had PLD. Therefore, previous studies and the current one show that the presence of atypical kidney cysts and the absence of hepatic cysts, despite kidney enlargement, can be a characteristic of polycystic kidneys caused by the *IFT140* variant. In terms of kidney function, patients with pathogenic *IFT140* variants had the highest eGFR (Table 2 and Figure 3a). In the previous study on genetic analysis of ADPKD-diagnosed families, patients with *IFT140* pathogenic variants had a milder chronic kidney disease course than those with *PKD2* pathogenic variants.^6^ These results suggest that patients with *IFT140*-related polycystic kidneys are likely underdiagnosed due to their relatively high eGFR and atypical kidney cysts. Therefore, the high proportion of *IFT140* pathogenic variants in cases without a positive family history of polycystic kidneys may be attributed not only to novel variants but also to the possibility that their parents were not diagnosed due to their milder phenotype.

Senum *et al.*^6^ reported that in 1 family, 2 individuals carrying a familial *IFT140* pathogenic variant exhibited no kidney cysts, as detected by ultrasound, at the age of 53 and 40 years. Furthermore, Chang *et al.*^23^ identified loss-of-function variants in *IFT140* in 205 of 112,392 unrelated participants who underwent exome analysis. However, only 5 of them (2.5%) were diagnosed with ADPKD according to the International Classification of Diseases, Ninth Revision or the International Statistical Classification of Diseases and Related Health Problems, Tenth Revision.^23^ Because kidney cysts may appear later in life, genotype–phenotype associations depend on the age of participants in each study cohort. Furthermore, patients with pathogenic variants in *IFT140* may not have been diagnosed with ADPKD because of their mild phenotype. However, these findings suggest that the penetrance of *IFT140* may be lower than that of *PKD1* or *PKD2* in patients with polycystic kidney disease.

In our study, some patients with a monoallelic *IFT140* loss-of-function variant had asymmetric kidney cysts (Patient ID. 723. [Figure 2b]). In addition, because the kidney cysts are large, some patients experienced mild kidney dysfunction despite having increased kidney volume (Patient ID. 942 [Figure 2d] and Patient ID. 1558 [Figure 2f]). Senum *et al.*^6^ reported that patients with *IFT140* pathogenic variants often had enlarged kidneys with a few large cysts, sometimes resulting in asymmetry. Because only a small number of cysts account for most of the TKV, it could be difficult to use TKV to predict kidney prognosis or to determine the suitability of tolvaptan treatment in patients with polycystic kidney disease caused by *IFT140*.

Unlike patients with a positive family history of polycystic kidneys, there are no definitive imaging findings that unequivocally diagnose ADPKD in patients without a positive family history of polycystic kidneys. Thus, it is crucial to differentiate ADPKD from other inherited kidney cystic diseases.^31^^,^^32^ In our study, 7 out of 157 patients had pathogenic variants in cystic kidney disease-related genes, excluding *IFT140*, *PKD1*, and *PKD2*. Three patients had HNF1B pathogenic variants, 2 patients had pathogenic variants in *PKHD1*, and 1 patient each had pathogenic variants in *OFD1* and *NPHP4* (Figure 1). Hepatocyte nuclear factor-1β, a DNA-binding transcription factor, is crucial for normal kidney development.^33^ Because hepatocyte nuclear factor-1β directly controls *PKD2* transcription, the cystic kidney disease seen in patients with *HNF1B* variants mirrors ADPKD. Furthermore, *HNF1B* variants have been detected in some individuals initially diagnosed with ADPKD but without *PKD1* and *PKD2* variants.^33^ *PKHD1* is the causative gene of ARPKD. ARPKD is generally considered the infantile type of polycystic kidneys, and a small proportion of patients are older children or teenagers.^34^ However, ARPKD is rarely diagnosed in adults.^34^^,^^35^ OFD1 is a rare X-linked dominant ciliopathy associated with congenital malformations such as cleft palate, tongue lobulation, cognitive impairment, and digital anomalies. Up to 50% of patients with multicystic kidney disease exhibit numerous large kidney cysts, mimicking ADPKD.^36^ Our results suggest that comprehensive genetic analysis is beneficial for patients with polycystic kidneys who did not have a family history of polycystic kidneys.

This study has some limitations. First, the genetic analysis was conducted solely on probands. Because the median patient age in this study was over 50 years, we could not obtain samples from their parents. The mild phenotype of polycystic kidneys due to *IFT140* may cause parental kidney disease to be overlooked. However, samples from some family members may be available. We plan to conduct further analysis in the future. Second, despite our gene panel covering most genes linked to inherited kidney cystic disease, it did not include recently identified genes such as *DNAJB11*,^37^ *ALG5*,^38^ and *TSC1*.^39^ Third, although we conducted copy number variation analysis using next-generation sequencing data, we could not perform multiplex ligation-dependent amplification or array-based comparative genome hybridization. In addition, Sanger sequencing of exon 1 of the *PKD1* gene, which has a high guanine-cytosine content, was not performed. Therefore, the proportion of the *PKD1* or *PKD2* pathogenic variants might have been underestimated. Finally, this study included patients whose parents did not have polycystic kidneys who presented with 5 or more cysts in each kidney. Although, this is a common issue when diagnosing patients with polycystic kidneys who did not have a family history of polycystic kidneys, these patient selection criteria may miss patients with unilateral or asymmetric or focal polycystic kidneys. In addition, elderly patients aged 60 years and older may present with acquired or age-related cystic kidney disease. Despite these limitations, to the best of our knowledge, this study is the first focus on cases of polycystic kidneys without a positive family history of polycystic kidneys only and to identify the subclinical rate of *IFT140*.

In conclusion, at least 4.5% of adult patients with polycystic kidneys whose parents do not have evident polycystic kidneys have monoallelic loss-of-function *IFT140* variants. Adult patients with polycystic kidneys whose parents do not have evident polycystic kidneys may have more *IFT140* pathogenic variants than those with a family history of polycystic kidneys. Patients with *IFT140*-related polycystic kidneys are likely to be underdiagnosed because of relatively high eGFR and atypical kidney cysts. The proportion of *PKD1* or *PKD2* pathogenic variants in patients without a positive family history of polycystic kidneys is low, and the presence of *IFT140* pathogenic variants is significant.

## Disclosure

TM belonged to an endowed department sponsored by Otsuka Pharmaceutical Co., Chugai Pharmaceutical Co., Kyowa Kirin Co., and JMS Co. and received honoraria for lectures from Otsuka Pharmaceutical Co. All the other authors declared no competing interests.

## Supplementary Material

Supplementary File (PDF) **Supplementary Methods. Supplementary References. Table S1.** Clinical features of patients with PKD1 or PKD2 pathogenic variants. **Table S2.** Clinical features of patients with other pathogenic variants. **Table S3.** Clinical features of patients with variants of unknown significance in PKD1 or PKD2. **Table S4.** Clinical features of patients with variants of unknown significance in other genes.
