# Supplementary material for: Importance of IFT140 in Patients with Polycystic Kidney Disease Without a Family History
Source: Kidney Int Rep. 2024 Jul 16;9(9):2685–94. doi: 10.1016/j.ekir.2024.06.021 (PMC11403091; doi:10.1016/j.ekir.2024.06.021)
Supplement: Supplementary File (PDF) — Supplementary Methods. Supplementary References. Table S1. Clinical features of patients with PKD1 or PKD2 pathogenic variants. Table S2. Clinical features of patients with other pathogenic variants. Table S3. Clinical features of patients with variants of unknown significance in PKD1 or PKD2. Table S4. Clinical features of patients with variants of unknown significance in other genes. [file mmc1.pdf]

Supplementary Methods

Capture-based sequencing

RNA capture probes for the coding regions of either 69 or 92 genes were designed using Agilent's SureDesign service (https://earray.chem.agilent.com/suredesign/home.htm). Pooled and barcoded libraries for next-generation sequencing were prepared using SureSelect QXT kits (Agilent Technologies, Santa Clara, California) as per the manufacturer's protocol. The prepared libraries were sequenced using 150-bp SE reads on Illumina MiSeq sequencers. Candidate variants were filtered as previously described[S1]. We initially excluded single nucleotide variants (SNVs) with allele frequencies greater than 0.01 in any population from the following databases: gnomAD[S2](https://gnomad.broadinstitute.org/gene), National Heart, Lung, and Blood Institute Exome Sequencing Project exome variant server dataset ESP6500[S3](http://evs.gs.washington.edu/EVS/), 1000 Genomes catalog(http://browser.1000genomes.org/index.html), Human Genetic Variation Database[S4](HGVD: http://www.genome.med.kyoto-u.ac.jp/SnpDB/), and the allele frequency panel of 8,380 Japanese individuals from The Tohoku Medical Megabank Organization(https://jmorp.megabank.tohoku.ac.jp) [S5]. We excluded SNVs of "LOW" impact severities as defined by the GEMINI software[S6]. This includes functional predictions such as "synonymous_coding," "intergenic," "upstream," "UTR," "intron," etc. We interpreted the significance of the variants using Polyphen2[S7], SIFT[S8], CADD[S9], MCAP[S10], and GERP conservation scores[S11]. The literature available in the Human Gene Mutation Database Pro[S12] was reviewed to assess the evidence on the pathogenicity of previously reported variants. The methods were detailed in our previous reports[S13, S14].

Supplementary Table S1. Clinical features of patients with *PKD1* or *PKD2* pathogenic variants.

Patient ID.	Age[a], year	Sex	HT	eGFR[a], ml/min/ 1.73m^2	TKV[a], ml	Gene[b]	Variant	Zygosity	gnomAD[c]	ToMMo 8.3K[d]	CADD[e]	ACMG	Reports
548	61	M	+	KF	4603	*PKD1*	c.11692_11693del:p.Ser3898fs	het.	none	none	33	P	none
585	43	M	+	22.3	1436	*PKD1*	c.2494dup:p.Arg832fs	het.	none	none	22.7	P	S15–18
600	76	M	+	24.0	975	*PKD1*	c.7546C>T:p.Arg2516Cys	het.	none	none	32	LP	S16, S17, S19–23
601	58	F	+	22.0	2056	*PKD1*	c.11816G>A:p.Trp3939Ter	het.	none	none	48	P	S13, S22
621	42	M	−	50.7	792	*PKD1*	c.3607C>T:p.Gln1203Ter	het.	none	none	34	P	S17, S18, S24
631[f]	46	M	+	92.6	854	*PKD2*<br>*PKD1*	c.1249C>T:p.Arg417Ter<br>c.10904C>A:p.Ala3635Asp	het.<br>het.	0.00003<br>0.00002	none<br>0.004	36<br>18.3	P<br>VUS	S16–18, S22, S25–29<br>S14, S17
632	54	M	+	KF	3449	*PKD2*	c.239C>A:p.Ser80Ter	het.	0.000009	none	35	LP	S13, S17, S22
634	49	M	+	62.8	1460	*PKD2*<br>*PKD1*	c.933T>G:p.Tyr311Ter<br>c.848C>T:p.Pro283Leu	het.<br>het.	none<br>0.000005	none<br>none	24.2<br>16.6	LP<br>VUS	S30<br>S13
648	45	F	+	13.8	2482	*PKD1*	c.1522T>G:p.Cys508Gly	het.	none	none	24.2	LP	S13, S22
654[g]	40	M	+	14.8	3085	*PKD1*<br>*PKD1*	c.8631delC:p.Asn2878fs<br>c.6680A>T:p.His2227Leu	het.<br>het.	none<br>0.000004	none<br>none	32<br>25.0	P<br>VUS	S13, S22<br>none
655	36	F	+	80.8	1859	*PKD1*<br>*PKD2*	c.1287G>A:p.Trp429Ter<br>c.2668G>A:p.Glu890Lys	het.<br>het.	none<br>0.00002	none<br>0.004	39<br>29.3	P<br>VUS	S13<br>S17, S22, S31, S32
743	47	M	+	25.5	2030	*PKD1*	c.12419G>A:p.Trp4140Ter	het.	none	none	56	P	none
758	51	F	+	81.1	1166	*PKD1*	c.11614G>C:p.Glu3872Gln	het.	none	none	24.8	LP	S20, S33, S34
764	51	M	+	19.4	6283	*PKD1*	c.11569_11572dup:p.Ser3858fs	het.	none	none	33	LP	S13, S22
773	73	M	−	52.1	1630	*PKD1*<br>*PKD2*	c.6733_6740del:p.Ile2245fs<br>c.2668G>A:p.Glu890Lys	het.<br>het.	none<br>0.00002	none<br>0.004	32<br>29.3	LP<br>VUS	S13, S22<br>S17, S22, S31, S32
774	52	F	+	51.8	920	*PKD1*	c.6472C>T:p.Gln2158Ter	het.	none	none	49	P	S22, S27, S35–37
834	47	F	+	KF	6488	*PKD1*	c.1687C>T:p.Gln563Ter	het.	none	none	35	P	S22, S27, S38, S39

837[f]	40	F	+	48.7	1609	*PKD1*	c.6727_6728del:p.Gln2243fs	het.	none	none	31	P	S34, S37, S40–43
838	52	M	+	25.3	5780	*PKD1*	c.5316dup:p.Thr1773fs	het.	none	none	21.8	LP	S13, S22
863	62	M	−	71.8	960	*PKD2*	c.878G>A:p.Trp293Ter	het.	none	none	38	P	S22
871	68	M	+	39.1	1041	*PKD2*	c.420del:p.Gly143fs	het.	0.00002	none	23	LP	S22
894	46	F	+	39.1	1312	*PKD1*<br>*IFT140*	c.6730_6731del:p.Ser2244fs<br>c.3602G>A:p.Arg1201His	het.<br>het.	none<br>0.00006	none<br>0.005	31<br>23.5	P<br>VUS	S44<br>none
952	68	M	+	19.3	1637	*PKD2*	c.2159dup:p.Asn720fs	het.	0.000008	none	32	P	S17, S22, S45–50
956	63	F	−	KF	1997	*PKD1*	c.1669_1670del:p.Leu557fs	het.	0.000005	none	20.2	LP	S17, S22
979	47	M	+	27.5	1619	*PKD1*	c.4292_4294delinsC:p.Val1431fs	het.	none	none	22.2	LP	none
1016	52	F	+	41.6	1512	*PKD2*<br>*PKD1*<br>*IFT140*	c.1390C>T:p.Arg464Ter<br>c.10748G>A:p.Gly3583Asp<br>c.1405C>A:p.Leu469Ile	het.<br>het.<br>het.	none<br>0.00001<br>0.000008	none<br>0.0004<br>0.0006	38<br>14.7<br>11.7	P<br>VUS<br>VUS	S17, S27, S28, S37, S51–53<br>none<br>none
1020	49	M	+	66.4	809	*PKD2*	c.2419C>T:p.Arg807Ter	het.	none	none	37	P	S18, S27, S28, S51, S54, S55
1021	59	M	+	4.4	2754	*PKD1*	c.12444+1G>A	het.	none	none	33	P	S56–58
1023	49	F	−	98.0	359	*PKD2*	c.1774C>T:p.Arg592Ter	het.	none	none	37	P	S28, S33, S58–60
1039	56	M	+	13.4	N/A	*PKD2*	c.916C>T:p.Arg306Ter	het.	0.000004	none	37	P	S16–18, S27, S37, S50, S52, S61–64
1097	55	F	N/A	20.8	2224	*PKD1*<br>*PKD1*	c.2085dup:p.Ala696fs<br>c.4967T>C:p.Val1656Ala	het.<br>het.	none<br>none	none<br>0.0001	16.4<br>22.8	P<br>VUS	S16, S17, S40, S65<br>S32
1147	52	F	N/A	64.1	598	*PKD2*	c.1249C>T:p.Arg417Ter	het.	0.00003	none	36	P	S16–18, S22, S25–29
1174	49	F	+	55.3	315	*PKD2*<br>*IFT140*	c.2407C>T:p.Arg803Ter<br>c.602A>C:p.Glu201Ala	het.<br>het.	0.00001<br>none	none<br>none	39<br>23.7	P<br>VUS	S17, S27, S28, S50, S66–69<br>none
1175	54	M	+	41.1	2315	*PKD1*<br>*IFT140*	c.8284_8295dup:p.Ile2762_Arg2765dup<br>c.3580C>G:p.Gln1194Glu	het.<br>het.	none<br>0.0001	none<br>0.0001	19.6<br>22.2	LP<br>VUS	S40<br>S70

1176[h]	52	M	+	54.6	2395	*PKD1*	c.9559_9561del:p.Asp3187del	het.	none	none	22.9	P	S71
						PKD1	c.7825A>G:p.Ile2609Val	het.	0.00003	0.001	17.0	VUS	none
1177	47	M	+	KF	7142	*PKD1*	c.6586C>T:p.Gln2196Ter	het.	none	none	47	P	S23, S72, S73
						PKD2	c.1255A>G:p.Thr419Ala	het.	0.00002	0.002	24.4	VUS	S32, S74
1235	70	F	N/A	KF	N/A	*PKD1*	c.8311G>A:p.Glu2771Lys	het.	none	none	29.5	LP	S16–18, S20, S25, S36, S40, S46, S50, S55, S75–80
1252	40	M	+	75.5	1467	*PKD1*	c.11555T>C:p.Leu3852Pro	het.	none	none	25.9	LP	S81
1332	35	M	−	55.3	951	*PKD1*	c.10745del:p.Pro3582fs	het.	none	none	32	LP	S17, S66, S82
1368	31	M	N/A	N/A	N/A	*PKD2*	c.958C>T:p.Arg320Ter	het.	0.000004	none	36	P	S16, S17, S27, S28, S36, S83, S84
1383	49	M	N/A	N/A	1058	*PKD2*	c.2419C>T:p.Arg807Ter	het.	none	none	37	P	S18, S27, S28, S51, S54, S55
1486	44	M	+	26.6	N/A	*PKD1*	c.1198C>T:p.Arg400Ter	het.	0.00001	none	33	P	S17, S27, S46, S85
1565	58	F	N/A	N/A	481	*PKD2*	c.973C>T:p.Arg325Ter	het.	0.00001	none	34	P	S27, S28, S46, S50, S60, S65, S76, S82, S86, S87
						PKD1	c.10904C>A:p.Ala3635Asp	het.	0.00002	0.004	18.3	VUS	S14, S17
						IFT140	c.4309G>A: p.Glu1437Lys	het.	0.0002	0.001	14.3	VUS	S17
1567	33	F	N/A	N/A	1472	*PKD1*	c.5201_5202del: p.Thr1734fs	het.	none	none	14.6	LP	none
1578	46	M	+	56.0	1359	*PKD1*	c.5968_5969del:p.Arg1990fs	het.	none	none	22.5	P	S33
						ALG8	c.323T>G:p.Phe108Cys	het.	none	none	23.2	VUS	none
1598	45	M	+	22.1	879	*PKD1*	c.10724G>A:p.Trp3575Ter	het.	none	none	52	P	S46
						PKD1	c.5957C>T:p.Thr1986Met	het.	0.0002	0.004	23.4	VUS	none
1626	31	F	+	81.6	1060	*PKD1*	c.11104C > T:p.Gln3702Ter	het.	none	none	42	P	S17, S27, S88
1628	31	M	−	100.7	1178	*PKD1*	c.8935_8937del:p.2979del	het.	0.00003	none	18.3	P	S17, S34, S76, S89
						PKD1	c.11053C>G:p.Leu3685Val	het.	none	none	23.0	VUS	none

						PKD2	c.2543G>A:p.Arg848Gln	het.	0.00006	0.002	25.3	VUS	none
1667	34	M	N/A	59.4	N/A	*PKD1*	c.12239G>A:p.Trp4080Ter	het.	none	none	47	P	[S33]
						PKD1	c.10904C>A:p.Ala3635Asp	het.	0.00002	0.004	18.3	VUS	[S14, S17]
1832	36	M	−	74.2	1162	*PKD1*	c.8284_8295del:p.Ile2762_Arg2765del	het.	none	none	21.5	LP	[S34, S90]
						PKD2	c.2638G>C:p.Val880Leu	het.	none	none	25	VUS	none
1849	40	M	+	29	N/A	*PKD1*	c.11153G>A:p.Arg3719Gln	het.	none	none	34	LP	[S25, S27, S37, S81, S91–93]

a at the time of genetic analysis

b The following NCBI reference sequences were used: *PKD1*, NM_001009944; *PKD2*, NM_000297; *IFT140*, NM_014714; *ALG8*, NM_024079.

c Genome Aggregation Database, v2.1.1[S2].

d Allele frequency panel of 8,380 Japanese individuals from The Tohoku Medical Megabank Organization[S5].

e Combined Annotation-Dependent Depletion phred score[S9].

f Patient's mother had simple kidney cyst.

g Patient's farther had simple kidney cyst.

h Patient's brother had simple kidney cyst.

eGFR, estimated glomerular filtration rate; TKV, total kidney volume; ACMG, American College of Medical Genetics and Genomics; M, male; F, female; HT, hypertension; N/A, not available; KF, kidney failure; het, heterozygous; P, pathogenic; VUS, variant of unknown significance; LP, likely pathogenic.

Supplementary Table S2. Clinical features of patients with other pathogenic variants.

Patient ID.	Age[a], year	Sex	HT	eGFR[a], ml/min/ $1.73m^2$	TKV[a], ml	Gene[b]	Variant	Zygosity	gnomAD[c]	ToMMo 8.3K[d]	CADD[e]	ACMG	Reports
478	25	M	+	33.2	791	*NPHP4*	c.2198G>A:p.Gly733Asp	het.	0.00007	0.003	24.3	LP	S13, S14, S22, S94–96
						NPHP4	c.2717G>A:p.Arg906His	het.	0.0002	0.009	9.4	VUS	S13, S14, S22, S49, S95, S96
500	37	M	−	25.4	202	*PKHD1*	c.1160A>T:p.Asn387Ile	het.	none	none	26.4	LP	S13, S22, S97, S98
						PKHD1	c.1396G>A:p.Gly466Arg	het.	none	0.0001	25.8	LP	S13, S22, S98, S99
562[f]	32	F	–	KF	1720	*OFD1*	c.508_509del:p.Asp170fs	het.	none	none	22.7	LP	S13, S22, S100, S101
1237	36	M	N/A	N/A	N/A	*PKHD1*	c.1242_1250del:p.Ala415_Ile417del	het.	none	0.0004	18.8	VUS	none
						PKHD1	c.5994_5998del: p.Gly1999fs	het.	none	none	33	LP	none
1354	58	M	+	25.3	N/A	*HNF1B*	Large deletion calculated approximately using CONTRA coordinates.	het.	N/A	N/A	N/A	P	-
1392	21	F	−	40.8	N/A	*HNF1B*	c.1211_1212del:p.Ser404fs	het.	none	none	34	LP	S102
1563	53	F	N/A	KF	601	*HNF1B*	c.107C>T:p.Ser36Phe	het.	0.0001	0.0002	23.4	LP	S103–105

a at the time of genetic analysis

b The following NCBI reference sequences were used: *NPHP4*, NM_015102; *PKHD1*, NM_170724; *OFD1*, NM_003611; *HNF1B*, NM_000458.

c Genome Aggregation Database, v2.1.1 [S2].

d Allele frequency panel of 8,380 Japanese individuals from The Tohoku Medical Megabank Organization [S5].

e Combined Annotation-Dependent Depletion phred score [S9].

f Patient's mother had simple kidney cyst.

eGFR, estimated glomerular filtration rate; TKV, total kidney volume; ACMG, American College of Medical Genetics and Genomics; M, male; F, female; N/A, not available; KF, kidney

failure; het, heterozygous; P, pathogenic; VUS, variant of unknown significance; LP, likely pathogenic.

Supplementary Table S3. Clinical features of patients with variants of unknown significance in *PKD1* or *PKD2*.

Patient ID.	Age[a], year	Sex	HT	eGFR[a], ml/min/1.73m^2	TKV[a], ml	Gene[b]	Variant	Zygosity	gnomAD[c]	ToMMo 8.3K[d]	CADD[e]	ACMG	Reports
515	78	M	+	23.1	858	*PKD1*	c.11711C>G:p.Ser3904Trp	het.	none	none	24.8	VUS	S20
517	36	F	+	KF	1944	*PKD1*	c.11102T>G:p.Leu3701Arg	het.	none	none	24.6	VUS	S13, S22
521	71	M	+	29.1	792	*PKD1*	c.5645C>T:p.Thr1882Met	het.	0.00006	0.0004	22.6	VUS	S13, S49
603	39	F	−	78.9	1594	*PKD2*	c.2668G>A:p.Glu890Lys	het.	0.00002	0.004	29.3	VUS	none
625	40	M	−	41.4	284	*PKD1*	c.9505C>T:p.Arg3169Trp	het.	0.00003	0.0001	28.6	VUS	S13, S22, S49
763	62	F	+	18.1	1567	*PKD1*	c.9586T>A:p.Phe3196Ile	het.	none	none	22.4	VUS	S13, S22
767	81	F	+	4.1	3014	*PKD1*	c.6796C>G:p.Arg2266Gly	het.	none	none	23.4	VUS	S13, S22
841	59	M	+	66.7	730	*PKD1*	c.7100C>T:p.Ser2367Phe	het.	0.0001	0.008	26.5	VUS	S22, S36
842[f]	73	F	+	KF	1900	*PKD1*	c.7381G>C:p.Ala2461Pro	het.	none	none	27.5	VUS	S13, S22
864	63	M	+	27.7	610	*PKD1*	c.3416T>A:p.Val1139Glu	het.	none	none	22.7	VUS	S22
967	45	M	+	36.6	1841	*PKD1*	c.6643C>T:p.Arg2215Trp	het.	0.000008	none	29.9	VUS	S17, S20, S22, S23, S106
968	39	F	−	87.6	518	*PKD1*	c.542T>A:p.Val181Asp	het.	none	none	18.3	VUS	S22
1160	42	F	+	44.2	N/A	*PKD1*	c.3701C>T:p.Ala1234Val	het.	0.00007	0.0004	10.2	VUS	none
1162	48	M	+	74.4	N/A	*PKD1*	c.3876C>A:p.Phe1292Leu	het.	0.000004	0.004	21.1	VUS	none
1170	47	M	+	98.0	694	*PKD2*	c.595G>A:p.Gly199Ser	het.	none	none	32	VUS	S107
						IFT140	c.4309G>A:p.Glu1437Lys	het.	0.0002	0.001	14.3	VUS	S108
1172	28	M	+	64.6	1065	*PKD1*	c.2155C>T:p.His719Tyr	het.	none	none	24.3	VUS	none
1224	62	M	+	47.1	611	*PKD1*	c.6285C>A:Asp2095Glu	het.	0.00005	0.0005	22.4	VUS	S17, S32
1271	69	M	+	41.4	N/A	*PKD1*	c.1261C>T:p.Arg421Cys	het.	none	none	27	VUS	S16, S109, S110

1325	35	M	+	14.1	N/A	*PKD1*	c.7825A>G:p.Ile2609Val	het.	0.00003	0.001	17.0	VUS	none
1487	30	F	+	81.1	400	*PKD1*	c.11453G>T:p.Gly3818Val	het.	none	none	25.3	VUS	S55
1566	50	M	N/A	N/A	475	*PKD1*	c.12026T>A:p.Val4009Glu	het.	0.00002	0.0007	24.2	VUS	none
1570	54	F	N/A	N/A	N/A	*PKD1*	c.2878G>A:p.Gly960Ser	het.	0.00001	none	24.7	VUS	S17, S41, S106
1601	67	M	+	30.7	404	*PKD1*	c.4507G>T:p.Gly1503Trp	het.	none	none	25.6	VUS	none
1680	22	F	−	70.5	671	*PKD1*	c.5992C>T:p.Arg1998Cys	het.	0.00002	none	23.2	VUS	none
1697	58	M	+	67.1	421	*PKD2*	c.1094C>T:p.Ala365Val	het.	0.000004	none	32	VUS	none

a at the time of genetic analysis

b The following NCBI reference sequences were used: *PKD1*, NM_001009944; *PKD2*, NM_000297; *IFT140*, NM_014714.

c Genome Aggregation Database, v2.1.1[S2].

d Allele frequency panel of 8,380 Japanese individuals from The Tohoku Medical Megabank Organization[S5].

e Combined Annotation-Dependent Depletion phred score[S9].

f Patient's child had polycystic kidneys.

eGFR, estimated glomerular filtration rate; TKV, total kidney volume; ACMG, American College of Medical Genetics and Genomics; M, male; F, female; HT, hypertension; N/A, not available; KF, kidney failure; het, heterozygous; P, pathogenic; VUS, variant of unknown significance; LP, likely pathogenic.

Supplementary Table S4. Clinical features of patients with variants of unknown significance in other genes.

Patient ID.	Age[a], year	Sex	HT	eGFR[a], ml/min/ 1.73m^2	TKV[a], ml	Gene[b]	Variant	Zygosity	gnomAD[c]	ToMMo 8.3K[e]	CADD[e]	ACMG	Reports
757	51	F	−	73.1	1140	*HNF1B*	c.313G>A:p.Glu105Lys	het.	0.0001	0.001	28.1	VUS	none
1168	63	F	+	30.7	1284	*INVS*	c.1390G>A:p.Ala464Thr	het.	none	none	25.7	VUS	none
						INVS	c.1943A>G:p.Asn648Ser	het.	0.0002	0.002	1.0	VUS	[S111–113]

a at the time of genetic analysis

b The following NCBI reference sequences were used: *INVS*, NM_015102; *HNF1B*, NM_000458.

c Genome Aggregation Database, v2.1.1[S2].

d Allele frequency panel of 8,380 Japanese individuals from The Tohoku Medical Megabank Organization [S5].

e Combined Annotation-Dependent Depletion phred score[S9].

eGFR, estimated glomerular filtration rate; TKV, total kidney volume; ACMG, American College of Medical Genetics and Genomics; M, male; F, female; HT, hypertension; N/A, not available; het, heterozygous; P, pathogenic; VUS, variant of unknown significance; LP, likely pathogenic.

Supplementary References

S1. Chong JX, Burrage LC, Beck AE, et al. Autosomal-Dominant Multiple Pterygium Syndrome Is Caused by Mutations in MYH3. *Am J Hum Genet*. 2015;96(5):841-849. doi:10.1016/j.ajhg.2015.04.004

S2. Karczewski KJ, Francioli LC, Tiao G, et al. The mutational constraint spectrum quantified from variation in 141,456 humans. *Nature*. 2020;581(7809):434-443. doi:10.1038/s41586-020-2308-7

S3. 1000 Genomes Project Consortium, Auton A, Brooks LD, et al. A global reference for human genetic variation. *Nature*. 2015;526(7571):68-74. doi:10.1038/nature15393

S4. Higasa K, Miyake N, Yoshimura J, et al. Human genetic variation database, a reference database of genetic variations in the Japanese population. *J Hum Genet*. 2016;61(6):547-553. doi:10.1038/jhg.2016.12

S5. Tadaka S, Hishinuma E, Komaki S, et al. jMorp updates in 2020: large enhancement of multi-omics data resources on the general Japanese population. *Nucleic Acids Res*. 2021;49(D1):D536-D544. doi:10.1093/nar/gkaa1034

S6. Paila U, Chapman BA, Kirchner R, Quinlan AR. GEMINI: integrative exploration of genetic variation and genome annotations. *PLoS Comput Biol*. 2013;9(7):e1003153. doi:10.1371/journal.pcbi.1003153

S7. Adzhubei IA, Schmidt S, Peshkin L, et al. A method and server for predicting damaging missense mutations. *Nat Methods*. 2010;7(4):248-249. doi:10.1038/nmeth0410-248

S8. Kumar P, Henikoff S, Ng PC. Predicting the effects of coding non-synonymous variants on protein function using the SIFT algorithm. *Nat Protoc*. 2009;4(7):1073-1081. doi:10.1038/nprot.2009.86

S9. Kircher M, Witten DM, Jain P, O'Roak BJ, Cooper GM, Shendure J. A general framework for estimating the relative pathogenicity of human genetic variants. *Nat Genet*. 2014;46(3):310-315. doi:10.1038/ng.2892

S10. Jagadeesh KA, Wenger AM, Berger MJ, et al. M-CAP eliminates a majority of variants of uncertain significance in clinical exomes at high sensitivity. *Nat Genet*. 2016;48(12):1581-1586. doi:10.1038/ng.3703

S11. Davydov EV, Goode DL, Sirota M, Cooper GM, Sidow A, Batzoglou S. Identifying a high fraction of the human genome to be under selective constraint using GERP++. *PLoS Comput Biol*. 2010;6(12):e1001025. doi:10.1371/journal.pcbi.1001025

S12. Stenson PD, Mort M, Ball EV, et al. The Human Gene Mutation Database: towards a comprehensive repository of inherited mutation data for medical research, genetic diagnosis and next-generation sequencing studies. *Hum Genet*. 2017;136(6):665-677. doi:10.1007/s00439-017-1779-6

S13. Fujimaru T, Mori T, Sekine A, et al. Kidney enlargement and multiple liver cyst formation implicate mutations in PKD1/2 in adult sporadic polycystic kidney disease. *Clin Genet*. 2018;94(1):125-131.

doi:10.1111/cge.13249

S14. Mori T, Hosomichi K, Chiga M, et al. Comprehensive genetic testing approach for major inherited kidney diseases, using next-generation sequencing with a custom panel. *Clin Exp Nephrol*. 2017;21(1):63-75. doi:10.1007/s10157-016-1252-1

S15. Rossetti S, Hopp K, Sikkink RA, et al. Identification of gene mutations in autosomal dominant polycystic kidney disease through targeted resequencing. *J Am Soc Nephrol*. 2012;23(5):915-933. doi:10.1681/ASN.2011101032

S16. Groopman EE, Marasa M, Cameron-Christie S, et al. Diagnostic Utility of Exome Sequencing for Kidney Disease. *N Engl J Med*. 2019;380(2):142-151. doi:10.1056/NEJMoa1806891

S17. Kim H, Park HC, Ryu H, et al. Genetic Characteristics of Korean Patients with Autosomal Dominant Polycystic Kidney Disease by Targeted Exome Sequencing. *Sci Rep*. 2019;9(1):16952. doi:10.1038/s41598-019-52474-1

S18. Domingo-Gallego A, Pybus M, Bullich G, et al. Clinical utility of genetic testing in early-onset kidney disease: seven genes are the main players. *Nephrol Dial Transplant*. 2022;37(4):687-696. doi:10.1093/ndt/gfab019

S19. Garcia-Gonzalez MA, Jones JG, Allen SK, et al. Evaluating the clinical utility of a molecular genetic test for polycystic kidney disease. *Mol Genet Metab*. 2007;92(1-2):160-167. doi:10.1016/j.ymgme.2007.05.004

S20. Cornec-Le Gall E, Audrézet MP, Chen JM, et al. Type of PKD1 mutation influences renal outcome in ADPKD. *J Am Soc Nephrol*. 2013;24(6):1006-1013. doi:10.1681/ASN.2012070650

S21. Helbig KL, Farwell Hagman KD, Shinde DN, et al. Diagnostic exome sequencing provides a molecular diagnosis for a significant proportion of patients with epilepsy. *Genet Med*. 2016;18(9):898-905. doi:10.1038/gim.2015.186

S22. Sekine A, Fujimaru T, Hoshino J, et al. Genotype-Clinical Correlations in Polycystic Kidney Disease with No Apparent Family History. *Am J Nephrol*. 2019;49(3):233-240. doi:10.1159/000497444

S23. Nielsen ML, Lildballe DL, Rasmussen M, Bojesen A, Birn H, Sunde L. Clinical genetic diagnostics in Danish autosomal dominant polycystic kidney disease patients reveal possible founder variants. *Eur J Med Genet*. 2021;64(4):104183. doi:10.1016/j.ejmg.2021.104183

S24. Cornec-Le Gall E, Audrézet MP, Rousseau A, et al. The PROPKD Score: A New Algorithm to Predict Renal Survival in Autosomal Dominant Polycystic Kidney Disease. *J Am Soc Nephrol*. 2016;27(3):942-951. doi:10.1681/ASN.2015010016

S25. Chang AR, Moore BS, Luo JZ, et al. Exome Sequencing of a Clinical Population for Autosomal Dominant Polycystic Kidney Disease. *JAMA*. 2022;328(24):2412-2421. doi:10.1001/jama.2022.22847

S26. Pei Y, He N, Wang K, et al. A spectrum of mutations in the polycystic kidney disease-2 (PKD2) gene

from eight Canadian kindreds. *J Am Soc Nephrol*. 1998;9(10):1853-1860. doi:10.1681/ASN.V9101853

S27. Xiong HY, Alipanahi B, Lee LJ, et al. RNA splicing. The human splicing code reveals new insights into the genetic determinants of disease. *Science*. 2015;347(6218):1254806. doi:10.1126/science.1254806

S28. Xu D, Bian R, Tuo S, et al. PKD2 gene variants in Chinese patients with autosomal dominant polycystic kidney disease. *Clin Genet*. 2021;100(3):340-347. doi:10.1111/cge.14008

S29. Zacchia M, Blanco FDV, Trepiccione F, et al. Nephroplex: a kidney-focused NGS panel highlights the challenges of PKD1 sequencing and identifies a founder BBS4 mutation. *J Nephrol*. 2021;34(6):1855-1874. doi:10.1007/s40620-021-01048-4

S30. Kinoshita M, Higashihara E, Kawano H, et al. Technical Evaluation: Identification of Pathogenic Mutations in PKD1 and PKD2 in Patients with Autosomal Dominant Polycystic Kidney Disease by Next-Generation Sequencing and Use of a Comprehensive New Classification System. *PLoS One*. 2016;11(11):e0166288. doi:10.1371/journal.pone.0166288

S31. Kurashige M, Hanaoka K, Imamura M, et al. A comprehensive search for mutations in the PKD1 and PKD2 in Japanese subjects with autosomal dominant polycystic kidney disease. *Clin Genet*. 2015;87(3):266-272. doi:10.1111/cge.12372

S32. Hirota K, Akagawa H, Onda H, Yoneyama T, Kawamata T, Kasuya H. Association of Rare Nonsynonymous Variants in PKD1 and PKD2 with Familial Intracranial Aneurysms in a Japanese Population. *J Stroke Cerebrovasc Dis*. 2016;25(12):2900-2906. doi:10.1016/j.jstrokecerebrovasdis.2016.08.002

S33. Audrézet MP, Cornec-Le Gall E, Chen JM, et al. Autosomal dominant polycystic kidney disease: comprehensive mutation analysis of PKD1 and PKD2 in 700 unrelated patients. *Hum Mutat*. 2012;33(8):1239-1250. doi:10.1002/humu.22103

S34. Benson KA, Murray SL, Senum SR, et al. The genetic landscape of polycystic kidney disease in Ireland. *Eur J Hum Genet*. 2021;29(5):827-838. doi:10.1038/s41431-020-00806-5

S35. Rossetti S, Chauveau D, Walker D, et al. A complete mutation screen of the ADPKD genes by DHPLC. *Kidney Int*. 2002;61(5):1588-1599. doi:10.1046/j.1523-1755.2002.00326.x

S36. Jin M, Xie Y, Chen Z, et al. System analysis of gene mutations and clinical phenotype in Chinese patients with autosomal-dominant polycystic kidney disease. *Sci Rep*. 2016;6:35945. doi:10.1038/srep35945

S37. Xu D, Ma Y, Gu X, et al. Novel Mutations in the PKD1 and PKD2 Genes of Chinese Patients with Autosomal Dominant Polycystic Kidney Disease. *Kidney Blood Press Res*. 2018;43(2):297-309. doi:10.1159/000487899

S38. McCluskey M, Schiavello T, Hunter M, et al. Mutation detection in the duplicated region of the polycystic kidney disease 1 (PKD1) gene in PKD1-linked Australian families. *Hum Mutat*. 2002;19(3):240-250. doi:10.1002/humu.10045

S39. Zamani M, Seifi T, Sedighzadeh S, et al. Whole-Exome Sequencing Application for Genetic Diagnosis of Kidney Diseases: A Study from Southwest of Iran. *Kidney360*. 2021;2(5):873-877. doi:10.34067/KID.0006902020

S40. Rossetti S, Strmecki L, Gamble V, et al. Mutation analysis of the entire PKD1 gene: genetic and diagnostic implications. *Am J Hum Genet*. 2001;68(1):46-63. doi:10.1086/316939

S41. Durkie M, Chong J, Valluru MK, Harris PC, Ong ACM. Biallelic inheritance of hypomorphic PKD1 variants is highly prevalent in very early onset polycystic kidney disease. *Genet Med*. 2021;23(4):689-697. doi:10.1038/s41436-020-01026-4

S42. Basel-Salmon L, Ruhrman-Shahar N, Orenstein N, et al. When phenotype does not match genotype: importance of "real-time" refining of phenotypic information for exome data interpretation. *Genet Med*. 2021;23(1):215-221. doi:10.1038/s41436-020-00938-5

S43. Jurgens SJ, Choi SH, Morrill VN, et al. Analysis of rare genetic variation underlying cardiometabolic diseases and traits among 200,000 individuals in the UK Biobank. *Nat Genet*. 2022;54(3):240-250. doi:10.1038/s41588-021-01011-w

S44. Yu C, Yang Y, Zou L, et al. Identification of novel mutations in Chinese Hans with autosomal dominant polycystic kidney disease. *BMC Med Genet*. 2011;12:164. doi:10.1186/1471-2350-12-164

S45. Pei Y, Wang K, Kasenda M, et al. A novel frameshift mutation induced by an adenosine insertion in the polycystic kidney disease 2 (PKD2) gene. *Kidney Int*. 1998;53(5):1127-1132. doi:10.1046/j.1523-1755.1998.00890.x

S46. He WB, Xiao WJ, Tan YQ, et al. Novel mutations of PKD genes in Chinese patients suffering from autosomal dominant polycystic kidney disease and seeking assisted reproduction. *BMC Med Genet*. 2018;19(1):186. doi:10.1186/s12881-018-0693-7

S47. Lanktree MB, Guiard E, Li W, et al. Intrafamilial Variability of ADPKD. *Kidney Int Rep*. 2019;4(7):995-1003. doi:10.1016/j.ekir.2019.04.018

S48. Backman JD, Li AH, Marcketta A, et al. Exome sequencing and analysis of 454,787 UK Biobank participants. *Nature*. 2021;599(7886):628-634. doi:10.1038/s41586-021-04103-z

S49. Kars ME, Başak AN, Onat OE, et al. The genetic structure of the Turkish population reveals high levels of variation and admixture. *Proc Natl Acad Sci U S A*. 2021;118(36):e2026076118. doi:10.1073/pnas.2026076118

S50. Mallawaarachchi AC, Lundie B, Hort Y, et al. Genomic diagnostics in polycystic kidney disease: an assessment of real-world use of whole-genome sequencing. *Eur J Hum Genet*. 2021;29(5):760-770. doi:10.1038/s41431-020-00796-4

S51. Viribay M, Hayashi T, Tellería D, et al. Novel stop and frameshifting mutations in the autosomal dominant polycystic kidney disease 2 (PKD2) gene. *Hum Genet*. 1997;101(2):229-234.

doi:10.1007/s004390050621

S52. Zhang M, Liu S, Xia X, Cui Y, Li X. Identification of novel mutations and risk assessment of Han Chinese patients with autosomal dominant polycystic kidney disease. *Nephrology (Carlton)*. 2019;24(5):504-510. doi:10.1111/nep.13270

S53. Schon KR, Horvath R, Wei W, et al. Use of whole genome sequencing to determine genetic basis of suspected mitochondrial disorders: cohort study. *BMJ*. 2021;375:e066288. doi:10.1136/bmj-2021-066288

S54. Paavola J, Schliffke S, Rossetti S, et al. Polycystin-2 mutations lead to impaired calcium cycling in the heart and predispose to dilated cardiomyopathy. *J Mol Cell Cardiol*. 2013;58:199-208. doi:10.1016/j.yjmcc.2013.01.015

S55. Pandita S, Ramachandran V, Balakrishnan P, et al. Identification of PKD1 and PKD2 gene variants in a cohort of 125 Asian Indian patients of ADPKD. *J Hum Genet*. 2019;64(5):409-419. doi:10.1038/s10038-019-0582-8

S56. Yu C, Li J, Yuan Z, Liu S, Zou L. Two novel mutations affecting the same splice site of PKD1 correlate with different phenotypes in ADPKD. *Ren Fail*. 2014;36(5):687-693. doi:10.3109/0886022X.2014.890010

S57. Jayasinghe K, Stark Z, Kerr PG, et al. Clinical impact of genomic testing in patients with suspected monogenic kidney disease. *Genet Med*. 2021;23(1):183-191. doi:10.1038/s41436-020-00963-4

S58. Li W, Liu G, Zhao X, et al. Genetic testing, ultrasonography and preimplantation genetic testing of men with autosomal dominant polycystic kidney disease in Hunan, China. *Andrologia*. 2022;54(1):e14273. doi:10.1111/and.14273

S59. Zhang W, Han Q, Liu Z, Zhou W, Cao Q, Zhou W. Whole exome sequencing reveals a stop-gain mutation of PKD2 in an autosomal dominant polycystic kidney disease family complicated with aortic dissection. *BMC Med Genet*. 2018;19(1):19. doi:10.1186/s12881-018-0536-6

S60. Wang J, Yang H, Guo R, Sang X, Mao Y. Association of a novel PKHD1 mutation in a family with autosomal dominant polycystic liver disease. *Ann Transl Med*. 2021;9(2):120. doi:10.21037/atm-20-3318

S61. Veldhuisen B, Saris JJ, de Haij S, et al. A spectrum of mutations in the second gene for autosomal dominant polycystic kidney disease (PKD2). *Am J Hum Genet*. 1997;61(3):547-555. doi:10.1086/515497

S62. Riccio E, Migliaccio S, Santangelo M, Pisani A. Arterial aneurysms: autosomal dominant polycystic kidney disease, Marfan syndrome or both? *Clin Exp Nephrol*. 2014;18(4):672-673. doi:10.1007/s10157-013-0876-7

S63. Capalbo A, Valero RA, Jimenez-Almazan J, et al. Optimizing clinical exome design and parallel gene-

testing for recessive genetic conditions in preconception carrier screening: Translational research genomic data from 14,125 exomes. *PLoS Genet*. 2019;15(10):e1008409. doi:10.1371/journal.pgen.1008409

S64. Seltzsam S, Wang C, Zheng B, et al. Reverse phenotyping facilitates disease allele calling in exome sequencing of patients with CAKUT. *Genet Med*. 2022;24(2):307-318. doi:10.1016/j.gim.2021.09.010

S65. Elliott MD, James LC, Simms EL, et al. Mainstreaming Genetic Testing for Adult Patients With Autosomal Dominant Polycystic Kidney Disease. *Can J Kidney Health Dis*. 2021;8:20543581211055001. doi:10.1177/20543581211055001

S66. Mallawaarachchi AC, Hort Y, Cowley MJ, et al. Whole-genome sequencing overcomes pseudogene homology to diagnose autosomal dominant polycystic kidney disease. *Eur J Hum Genet*. 2016;24(11):1584-1590. doi:10.1038/ejhg.2016.48

S67. Deltas CC. Mutations of the human polycystic kidney disease 2 (PKD2) gene. *Hum Mutat*. 2001;18(1):13-24. doi:10.1002/humu.1145

S68. Peces R, Mena R, Martín Y, et al. Co-occurrence of neurofibromatosis type 1 and optic nerve gliomas with autosomal dominant polycystic kidney disease type 2. *Mol Genet Genomic Med*. 2020;8(8):e1321. doi:10.1002/mgg3.1321

S69. Hsieh PF, Liu SY, Chen CH, Chen PL, Tang SC, Jeng JS. Genetic analysis of a family presenting with coexisting cerebral cavernous malformations and polycystic kidney disease. *J Formos Med Assoc*. 2022;121(11):2331-2337. doi:10.1016/j.jfma.2022.03.010

S70. Gao FJ, Li JK, Chen H, et al. Genetic and Clinical Findings in a Large Cohort of Chinese Patients with Suspected Retinitis Pigmentosa. *Ophthalmology*. 2019;126(11):1549-1556. doi:10.1016/j.ophtha.2019.04.038

S71. Carrera P, Calzavara S, Magistroni R, et al. Deciphering Variability of PKD1 and PKD2 in an Italian Cohort of 643 Patients with Autosomal Dominant Polycystic Kidney Disease (ADPKD). *Sci Rep*. 2016;6:30850. doi:10.1038/srep30850

S72. Obeidova L, Elisakova V, Stekrova J, et al. Novel mutations of PKD genes in the Czech population with autosomal dominant polycystic kidney disease. *BMC Med Genet*. 2014;15:41. doi:10.1186/1471-2350-15-41

S73. Elisakova V, Merta M, Reiterova J, et al. Bilineal inheritance of pathogenic PKD1 and PKD2 variants in a Czech family with autosomal dominant polycystic kidney disease - a case report. *BMC Nephrol*. 2018;19(1):163. doi:10.1186/s12882-018-0978-2

S74. Chung W, Kim H, Hwang YH, et al. PKD2 gene mutation analysis in Korean autosomal dominant polycystic kidney disease patients using two-dimensional gene scanning. *Clin Genet*. 2006;70(6):502-508. doi:10.1111/j.1399-0004.2006.00721.x

S75. Liu B, Chen SC, Yang YM, et al. Identification of novel PKD1 and PKD2 mutations in a Chinese population with autosomal dominant polycystic kidney disease. *Sci Rep*. 2015;5:17468. doi:10.1038/srep17468

S76. Kasap Demir B, Mutlubaş F, Soyaltın E, et al. Demographic and clinical characteristics of children with autosomal dominant polycystic kidney disease: a single center experience. *Turk J Med Sci*. 2021;51(2):772-777. doi:10.3906/sag-2009-79

S77. Münch J, Kirschner KM, Schlee H, et al. Autosomal dominant polycystic kidney disease in absence of renal cyst formation illustrates genetic interaction between WT1 and PKD1. *J Med Genet*. Published online May 2020:jmedgenet-2019-106633. doi:10.1136/jmedgenet-2019-106633

S78. Vaisitti T, Sorbini M, Callegari M, et al. Clinical exome sequencing is a powerful tool in the diagnostic flow of monogenic kidney diseases: an Italian experience. *J Nephrol*. 2021;34(5):1767-1781. doi:10.1007/s40620-020-00898-8

S79. Hu HY, Zhang J, Qiu W, et al. Comprehensive strategy improves the genetic diagnosis of different polycystic kidney diseases. *J Cell Mol Med*. 2021;25(13):6318-6332. doi:10.1111/jcmm.16608

S80. Wang H, Dai S, Zhang J, et al. Analysis of mutations in six Chinese families with autosomal dominant polycystic kidney disease. *Am J Transl Res*. 2020;12(12):8123-8136.

S81. Aguiari G, Savelli S, Garbo M, et al. Novel splicing and missense mutations in autosomal dominant polycystic kidney disease 1 (PKD1) gene: expression of mutated genes. *Hum Mutat*. 2000;16(5):444-445. doi:10.1002/1098-1004(200011)16:5<444::AID-HUMU11>3.0.CO;2-C

S82. Rossetti S, Consugar MB, Chapman AB, et al. Comprehensive molecular diagnostics in autosomal dominant polycystic kidney disease. *J Am Soc Nephrol*. 2007;18(7):2143-2160. doi:10.1681/ASN.2006121387

S83. Reynolds DM, Hayashi T, Cai Y, et al. Aberrant splicing in the PKD2 gene as a cause of polycystic kidney disease. *J Am Soc Nephrol*. 1999;10(11):2342-2351. doi:10.1681/ASN.V10112342

S84. Moriyama T, Nakayama Y, Soejima M, et al. Effect of tolvaptan on renal involvement in patients with autosomal dominant polycystic kidney disease according to different gene mutations. *Clin Exp Nephrol*. 2021;25(3):251-260. doi:10.1007/s10157-020-01988-4

S85. Peters DJ, Ariyurek Y, van Dijk M, Breuning MH. Mutation detection for exons 2 to 10 of the polycystic kidney disease 1 (PKD1)-gene by DGGE. *Eur J Hum Genet*. 2001;9(12):957-960. doi:10.1038/sj.ejhg.5200756

S86. Oka M, Mochizuki T, Kobayashi S. A novel mutation of the PKD2 gene in a Japanese patient with autosomal dominant polycystic kidney disease and complete situs inversus. *Am J Kidney Dis*. 2014;64(4):660. doi:10.1053/j.ajkd.2014.05.023

S87. Raj S, Singh RG, Das P. Mutational screening of PKD2 gene in the north Indian polycystic kidney disease

patients revealed 28 genetic variations. *J Genet*. 2017;96(6):885-893. doi:10.1007/s12041-017-0824-5

S88. Eo HS, Lee JG, Ahn C, et al. Three novel mutations of the PKD1 gene in Korean patients with autosomal dominant polycystic kidney disease. *Clin Genet*. 2002;62(2):169-174. doi:10.1034/j.1399-0004.2002.620211.x

S89. Bouba I, Koptides M, Mean R, et al. Novel PKD1 deletions and missense variants in a cohort of Hellenic polycystic kidney disease families. *Eur J Hum Genet*. 2001;9(9):677-684. doi:10.1038/sj.ejhg.5200696

S90. Neumann HPH, Bacher J, Nabulsi Z, et al. Adult patients with sporadic polycystic kidney disease: the importance of screening for mutations in the PKD1 and PKD2 genes. *Int Urol Nephrol*. 2012;44(6):1753-1762. doi:10.1007/s11255-012-0125-0

S91. Gonzalez-Paredes FJ, Ramos-Trujillo E, Claverie-Martin F. Defective pre-mRNA splicing in PKD1 due to presumed missense and synonymous mutations causing autosomal dominant polycystic disease. *Gene*. 2014;546(2):243-249. doi:10.1016/j.gene.2014.06.004

S92. Claverie-Martin F, Gonzalez-Paredes FJ, Ramos-Trujillo E. Splicing defects caused by exonic mutations in PKD1 as a new mechanism of pathogenesis in autosomal dominant polycystic kidney disease. *RNA Biol*. 2015;12(4):369-374. doi:10.1080/15476286.2015.1014291

S93. Bitarafan F, Garshasbi M. Molecular genetic analysis of polycystic kidney disease 1 and polycystic kidney disease 2 mutations in pedigrees with autosomal dominant polycystic kidney disease. *J Res Med Sci*. 2019;24:44. doi:10.4103/jrms.JRMS_835_18

S94. Li S, Liu S, Chen W, et al. A novel ZIC3 gene mutation identified in patients with heterotaxy and congenital heart disease. *Sci Rep*. 2018;8(1):12386. doi:10.1038/s41598-018-30204-3

S95. Takada D, Sekine A, Yabuuchi J, et al. Renal histology and MRI in a 25-year-old Japanese man with nephronophthisis 4. *Clin Nephrol*. 2018;89(3):223-228. doi:10.5414/CN109175

S96. König JC, Karsay R, Gerß J, et al. Refining Kidney Survival in 383 Genetically Characterized Patients With Nephronophthisis. *Kidney Int Rep*. 2022;7(9):2016-2028. doi:10.1016/j.ekir.2022.05.035

S97. Denamur E, Delezoide AL, Alberti C, et al. Genotype-phenotype correlations in fetuses and neonates with autosomal recessive polycystic kidney disease. *Kidney Int*. 2010;77(4):350-358. doi:10.1038/ki.2009.440

S98. Ito Y, Sekine A, Takada D, et al. Renal histology and MRI findings in a 37-year-old Japanese patient with autosomal recessive polycystic kidney disease. *Clin Nephrol*. 2017;88(11):292-297. doi:10.5414/CN109179

S99. Ishiko S, Morisada N, Kondo A, et al. Clinical features of autosomal recessive polycystic kidney disease in the Japanese population and analysis of splicing in PKHD1 gene for determination of phenotypes. *Clin Exp Nephrol*. 2022;26(2):140-153. doi:10.1007/s10157-021-02135-3

S100. Chetty-John S, Piwnica-Worms K, Bryant J, et al. Fibrocystic disease of liver and pancreas; under-

recognized features of the X-linked ciliopathy oral-facial-digital syndrome type 1 (OFD I). *Am J Med Genet A*. 2010;152A(10):2640-2645. doi:10.1002/ajmg.a.33666

S101. Bisschoff IJ, Zeschnigk C, Horn D, et al. Novel mutations including deletions of the entire OFD1 gene in 30 families with type 1 orofaciodigital syndrome: a study of the extensive clinical variability. *Hum Mutat*. 2013;34(1):237-247. doi:10.1002/humu.22224

S102. Nagano C, Morisada N, Nozu K, et al. Clinical characteristics of HNF1B-related disorders in a Japanese population. *Clin Exp Nephrol*. 2019;23(9):1119-1129. doi:10.1007/s10157-019-01747-0

S103. Yoshiuchi I, Yamagata K, Zhu Q, et al. Identification of a gain-of-function mutation in the HNF-1beta gene in a Japanese family with MODY. *Diabetologia*. 2002;45(1):154-155. doi:10.1007/s125-002-8259-5

S104. Wang C, Fang Q, Zhang R, Lin X, Xiang K. Scanning for MODY5 gene mutations in Chinese early onset or multiple affected diabetes pedigrees. *Acta Diabetol*. 2004;41(4):137-145. doi:10.1007/s00592-004-0157-8

S105. Liu JL, Wang XW, Liu CH, et al. Genetic spectrum of CAKUT and risk factors for kidney failure: a pediatric multicenter cohort study. *Nephrol Dial Transplant*. 2022;38(9):1981-1991. doi:10.1093/ndt/gfac338

S106. Neumann HPH, Jilg C, Bacher J, et al. Epidemiology of autosomal-dominant polycystic kidney disease: an in-depth clinical study for south-western Germany. *Nephrol Dial Transplant*. 2013;28(6):1472-1487. doi:10.1093/ndt/gfs551

S107. Bullich G, Domingo-Gallego A, Vargas I, et al. A kidney-disease gene panel allows a comprehensive genetic diagnosis of cystic and glomerular inherited kidney diseases. *Kidney Int*. 2018;94(2):363-371. doi:10.1016/j.kint.2018.02.027

S108. Seo GH, Kim T, Choi IH, et al. Diagnostic yield and clinical utility of whole exome sequencing using an automated variant prioritization system, EVIDENCE. *Clin Genet*. 2020;98(6):562-570. doi:10.1111/cge.13848

S109. Chang MY, Chen HM, Jenq CC, et al. Novel PKD1 and PKD2 mutations in Taiwanese patients with autosomal dominant polycystic kidney disease. *J Hum Genet*. 2013;58(11):720-727. doi:10.1038/jhg.2013.91

S110. Kim H, Kim HH, Chang CL, Song SH, Kim N. Novel PKD1 Mutations in Patients with Autosomal Dominant Polycystic Kidney Disease. *Lab Med*. 2021;52(2):174-180. doi:10.1093/labmed/lmaa047

S111. Tang X, Xu H, Shen Q, et al. Gene mutation and clinical analysis of nephronophthisis diagnosed using whole exome sequencing: Experience from China. *Clin Nephrol*. 2019;92(2):89-94. doi:10.5414/CN109571

S112. Rao J, Liu X, Mao J, et al. Genetic spectrum of renal disease for 1001 Chinese children based on a

multicenter registration system. *Clin Genet*. 2019;96(5):402-410. doi:10.1111/cge.13606

S113. Tang X, Liu C, Liu X, et al. Phenotype and genotype spectra of a Chinese cohort with nephronophthisis-related ciliopathy. *J Med Genet*. 2022;59(2):147-154. doi:10.1136/jmedgenet-2020-107184
